# Dicarbonyl stress, protein glycation and the unfolded protein response

**DOI:** 10.1007/s10719-021-09980-0

**Published:** 2021-03-01

**Authors:** Naila Rabbani, Mingzhan Xue, Paul J. Thornalley

**Affiliations:** 1 Department of Basic Medical Science, College of Medicine, QU Health, Qatar University, P.O. Box 2713, Doha, Qatar; 2 Biomedical & Pharmaceutical Research Unit, QU Health, Qatar University, P.O. Box 2713, Doha, Qatar; 3 Diabetes Research Center, Qatar Biomedical Research Institute, Hamad Bin Khalifa University, Qatar Foundation, P.O. Box 34110, Doha, Qatar

**Keywords:** Methylglyoxal, Glycation, Glyoxalase, Unfolded protein response, Heat shock response, Ubiquitin ligase, Low grade inflammation, Insulin resistance

## Abstract

The reactive dicarbonyl metabolite, methylglyoxal (MG), is increased in obesity and diabetes and is implicated in the development of insulin resistance, type 2 diabetes mellitus and vascular complications of diabetes. Dicarbonyl stress is the metabolic state of abnormal high MG concentration. MG is an arginine-directed glycating agent and precursor of the major advanced glycation endproduct, arginine-derived hydroimidazolone MG-H1. MG-H1 is often formed on protein surfaces and an uncharged hydrophobic residue, inducing protein structural distortion and misfolding. Recent studies indicate that dicarbonyl stress in human endothelial cells and fibroblasts *in vitro* induced a proteomic response consistent with activation of the unfolded protein response (UPR). The response included: increased abundance of heat shock proteins and ubiquitin ligases catalysing the removal of proteins with unshielded surface hydrophobic patches and formation of polyubiquitinated chains to encapsulate misfolded proteins; and increased low grade inflammation. Activation of the UPR is implicated in insulin resistance. An effective strategy to counter increased MG is inducing increased expression of glyoxalase-1 (Glo1). An optimized inducer of Glo1 expression, *trans*-resveratrol and hesperetin combination, normalized increased MG concentration, corrected insulin resistance and decreased low grade inflammation in overweight and obese subjects. We propose that dicarbonyl stress, through increased formation of MG-glycated proteins, may be an important physiological stimulus of the UPR and Glo1 inducers may provide a route to effective suppression and therapy. With further investigation and validation, this may provide key new insight into physiological activators of the UPR and association with dicarbonyl stress.

## Dicarbonyl stress

**Fig. 1:**
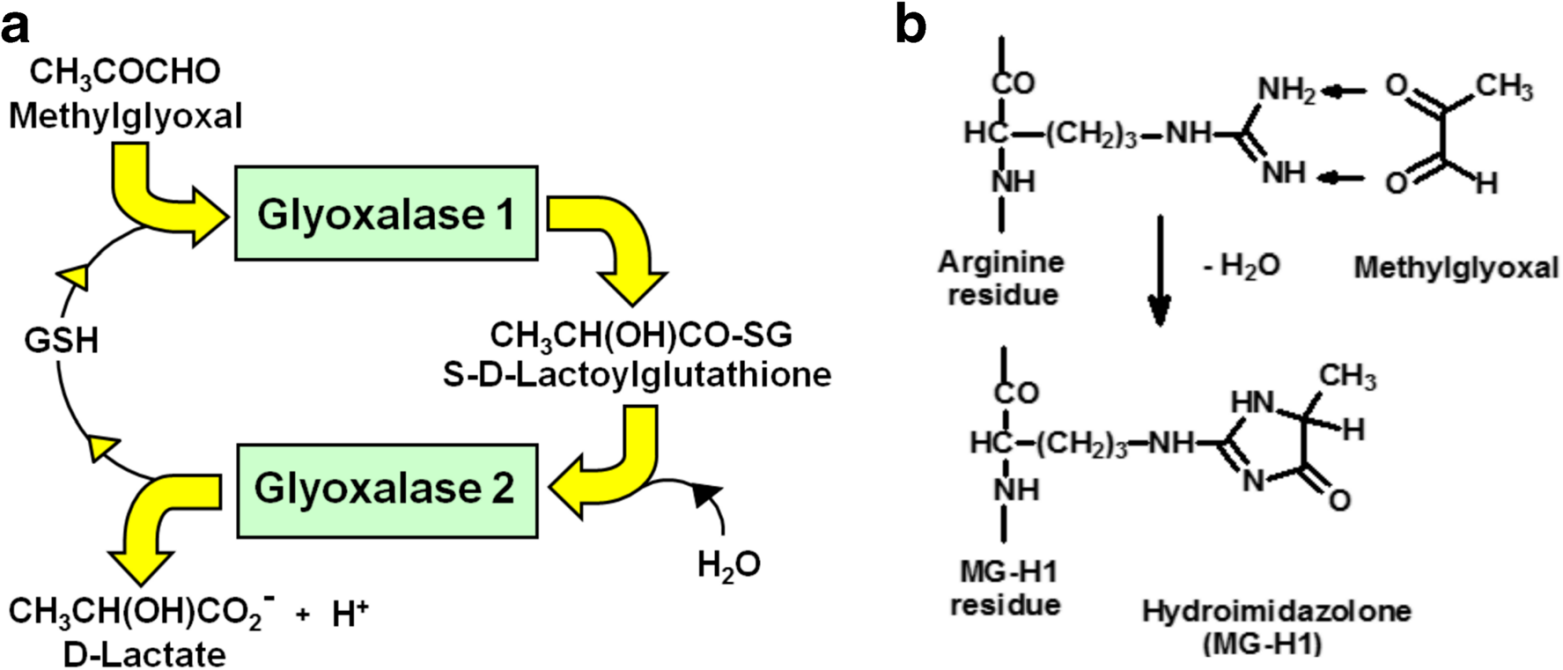
Major pathways of metabolism and glycation of methylglyoxal. **a** Metabolism of MG by the glyoxalase system. **b** Formation of hydroimidazolone MG-H1 from arginine residues

Dicarbonyl stress is the abnormal accumulation of dicarbonyl reactive metabolites leading to increased protein and DNA modification contributing to cell and tissue dysfunction in ageing and disease [1]. In the current context, we are considering the impact of protein glycation only. The major dicarbonyl reactive metabolite involved in protein glycation in physiological systems is methylglyoxal (MG). In mammalian metabolism, MG is mainly formed by the spontaneous, trace-level degradation of triosephosphates, glyceraldehyde-3-phosphate and dihydroxyacetonephosphate. Approximately 0.05–0.1 % flux of triosephosphates in glycolysis degrades to MG [2]. Cellular concentrations are low, typically 2–4 µM, but MG is a highly reactive metabolite and modifies proteins to form arginine-derived hydroimidazolone, MG-H1 – the major advanced glycation endproduct (AGE) formed in physiological systems [3]. The concentration of MG is maintained at these low levels by metabolism by glyoxalase 1 (Glo1) of the glyoxalase system. The glyoxalase system is comprised of two enzymes, Glo1 and glyoxalase 2 (Glo2) and a catalytic amount of reduced glutathione (GSH). Glo1 catalyses the conversion of the hemithioacetal formed spontaneously from MG and GSH to S-D-lactoylglutathione and Glo2 catalyses the onward conversion of this to D-lactate, reforming GSH consumed in the Glo1 catalysed step – Fig. 1a. The glyoxalase system is present in all cells and, by efficient metabolism of MG, suppresses the reaction of MG with protein to low tolerable levels [4]. The steady concentration of MG and related protein glycation is increased in aging, health disorders and disease where it likely contributes to decline in health and pathogenesis. This is evident in obesity, diabetes and vascular complications of diabetes (diabetic kidney disease, diabetic retinopathy, diabetic neuropathy and diabetic cardiovascular disease), cardiovascular disease and end stage renal disease – reviewed in [4–6]. Therapeutic agents in development to alleviate dicarbonyl stress, particularly inducers of Glo1 expression or “Glo1 inducers”, have shown promise in clinical trial [2, 5]. Recent research has implicated the accumulation of proteins glycated by MG with activation of the UPR and downstream inflammatory processes [7, 8]. In experimental studies, the UPR may be activated by inhibition of enzymatic N-linked glycosylation of proteins with tunicamycin – an inhibitor of UDP-N-acetylglucosamine: dolichyl-phosphate N-acetylglucosaminephosphotransferase [9]. However, this is not a physiological activator of the UPR. Glycation has been considered to be a contributory factor to protein misfolding and provide substrates for the UPR but the mechanisms remained unclear [10]. In this review, we describe recent advances and how MG glycation provides a challenge to protein homeostasis and physiological substrates for the UPR.

## Effect of methylglyoxal modification of proteins on protein folding

MG is an arginine-directed glycating agent, forming mainly MG-H1. Formation of MG-H1 produces loss of charge and an 18 % increase in molecular volume of the precursor arginine residue [11] – Fig. 1b. This produces loss of all electrostatic interactions. Physicochemical and molecular dynamic studies of proteins indicate arginine salt bridge interactions, particularly glu^−^/arg^+^ pairs, have a key role in kinetics and thermodynamic stability of protein folding, locking in to correct folding conformations [12]. Arginine residues are also common in cation-π interactions of the guanidinium sidechain cation with the aromatic π-electron distribution of tyrosine sidechains, providing intermolecular bonding at protein-protein interfaces [13]. All of these interactions are lost with modification by MG.

Regarding hydrophobicity, we applied the empirical prediction for hydrophobicity of amino acids from experimental measurements of the transfer energy (Etr) [14]. This suggests that Etr values for arginine and MG-H1 residues are 17.3 and 11.7, respectively, and related Eisenberg hydrophobicity *< H >* values are – 2.5 and − 0.6, respectively. This indicates formation of MG-H1produces a profound shift towards hydrophobicity; the closest uncharged amino acids with hydrophobicities similar to that predicted for MG-H1 are asparagine and glutamine with *< H >* values of − 0.78 and – 0.85, respectively. When MG modification occurs on arginine residues on the surface of proteins, as it typically does - often targeting residues with low microscopic pK_a_ which are activated towards modification [15, 16], there is creation of a hydrophobic residue on the surface of the protein substrate. This is different to the native protein structure where hydrophobic residues are often buried inside globular proteins or “shielded”. Surface unshielded hydrophobic residues are a key signature of misfolded proteins and a recognition feature leading to them being targetted for ubiquitination and degradation [17].

Initial studies of MG glycated proteins showed structural distortion and instability of modified proteins. In the assessment of the effect of MG modification on the structure and folding of proteins *in vivo*, it is important to reflect that proteins are minimally modified by MG with usually 1–5 % of protein modified [18]. Early studies of albumin and other proteins prepared *in vitro* with high extent of MG modification - often > 20 modifications per protein molecule – were a poor structural and functional model of proteins glycated by MG *in vivo* [19, 20]. For human serum albumin, low level modification by MG led to preferential modification of Arg-410 by MG to form a MG-H1 residue. Molecular graphics analysis of the MG-modified domain indicated that hydrogen bonding of this residue to Asn-391 was abolished and the proximate helical structure, helix 3Ah2, was distorted such that Arg-410 and Tyr-411 swung away from Asn-391, increasing the spatial separation of Asn-391 and Arg-410 [15]. Apolipoprotein B100 (apoB100), the major protein of low density lipoprotein (LDL) and very low density lipoprotein (VLDL) particles, is susceptible to MG modification *in vivo* [21]. Arg-18 was preferentially modified by MG in apoB100. A molecular graphics prediction of the structure of the MG-modified apoB100 was made based on structural similarity of the MG-modified domain of lipovitellin. This residue lies at the terminal focus of a lattice of seven parallel ß-strands. Conversion of this residue to MG-H1 disrupted and distorted interactions with all seven ß-strands and produced surface exposure of a proteoglycan binding domain [22]. Preferential sites of MG modification of apolipoprotein A-1 of high density lipoprotein (HDL) were Arg-27 and Arg-123. Conversion of Arg-27 to MG-H1 abolished the salt bridge interaction with Asp-29, and modification at Arg-123 to MG-H1 in helix 5 abolished the salt bridge interaction with Glu-120 of helix 4, with loss of interhelical bonding and related structural stability. MG modification of apolipoprotein A-1 was associated with decreased HDL stability and half-life [23]. We have listed and identified many other proteins susceptible to MG modification in our recent reports [7, 8, 16].

These characteristics of the arginine to MG-H1 transition and MG-modified proteins suggest that MG glycation may induce protein structural distortion and misfolding. Modification by glucose to form N-terminal and lysine sidechain fructosamine derivatives, N_α_-(1-deoxy-D-fructos-1-yl)amino acid and N_ε_-(1-deoxy-D-fructos-1-yl)lysine (FL), respectively, may induce much lower structural distortion and misfolding because the cationic charge and all electrostatic interactions are retained. The fructosyl moiety is also hydrophilic [11]. MG-modified proteins may therefore present a challenge to cell protein homeostasis and the protein quality control system, the UPR, may become activated for their removal.

## Activation of the unfolded protein response in dicarbonyl stress

Incubation of human aortal endothelial cells (HAECs) in high glucose concentration *in vitro* is an established model of endothelial cell dysfunction in hyperglycemia associated with diabetes. We found the cellular concentration of MG and MG-modified proteins was increased ca. 2-fold in HAECs incubated in 20 mM glucose, compared to 5 mM glucose normoglycemic control. MG concentration was increased in model hyperglycemia by increase in flux of glucose metabolism and concomitant increase in flux of MG formation, synergizing with decrease in Glo1 activity, linked to increased proteolysis [7]. To explore the mechanism of increased glucose metabolism in high glucose concentration – subsequently found to be due to glucose-induced stabilization of hexokinase-2 (HK2) to proteolysis, we analyzed and quantified the cytoplasmic proteome. The mean number of proteins identified in low and high glucose concentration was 1894. We found 331 proteins were upregulated with increased abundance in high glucose concentration cultures. There was increased of abundance of proteins in glycolysis and gluconeogenesis, which may be expected given the increased glucose metabolism through glycolysis and also into gluconeogenesis through metabolic channeling when HK2 is detached from mitochondria [7]. There was, however, also enrichment of proteins of the heat shock response – part of the UPR. Abundance increases in 8 heat shock proteins (HSPs) were recorded: HSPA8, HSP70 1A and 1B, HSPA1L, HSP105 kDa and HSPA9 (GRP75), HSPA5 (GRP78) and BAG5. These are chaperone proteins with expression increases by heat shock factor-1 (HSF-1) catalysing the refolding of proteins or recruiting ubiquitin E2 ligases for protein degradation by chaperone-assisted ubiquitin–proteasome pathway and autophagy. There were an additional 4 proteins of increased abundance of the heat shock response: replication protein A1 (RFA1), nucleoporin 214 kDa (NUP214), nuclear pore complex protein 133 kDa (NUP133) and nuclear pore complex protein 358 kDa (NUP358 – also known as RANBP2). RFA1 binds to HSF-1, assisting access to nucleosomal DNA by recruiting histone chaperone, facilitates chromatin transcription (FACT), which displaces the histone H2A-H2B dimer for transactivational activity of HSF-1 for increased HSP expression [24]. Increased nuclear pore protein abundance facilitates increased nuclear export of mRNA of HSPs for increased expression of these proteins in the response to proteotoxic stress [25] (Table 1).

**Table 1:** Proteins of regulation of HSF1-mediated heat shock response increased in HAECs in high glucose concentration *in vitro*

Protein (abbreviations)	Location	Function	Reference
Heat shock cognate 71 kDa, (HSPA8, HSP73)	Cytosol, nucleus	Chaperone-mediated autophagy. Binds with ubiquitin E2 ligase, CHIP	[68, 69]
Heat shock protein 70 kDa protein 1 (HSP701A, HSP1A, HSP72	Cytosol, nucleus	Chaperone. Binds CHIP, Ubc4/5 family of E2 enzymes and HUWE1. Chaperone-assisted ubiquitin–proteasome pathway and autophagy	[70]
Heat shock protein 70 kDa protein 2 (HSP701B, HSP1B)	Cytosol, nucleus	Chaperone. Binds CHIP, Ubc4/5 family of E2 enzymes and HUWE1. Chaperone-assisted ubiquitin–proteasome pathway and autophagy	[70]
Heat shock 70 kDa protein 1L (HSPA1L)	Cytosol, nucleus	Chaperone	[71]
Heat shock 105 kDa (HSP105, HSPH1, HSP110)	Cytosol, nucleus	Chaperone Interacts with HSC70 and HSP90. Chaperone-assisted ubiquitin–proteasome pathway and autophagy.	[72]
Glucose regulated protein 75 kDa (GRP75, HSPA9, MOT2, PBP74)	Mitochondria, cytosol, ER	Chaperone	[73]
Glucose regulated protein 78 kDa (GRP78, HSPA5, BIP, MIF2)	ER	Chaperone Activates activating transcription factor 6 (ATF6), PERK and IRE1α	[73]
Bcl-2- associated athanogene 5 (BAG5)	Cytosol, nucleus	Chaperone. CHIP Hsp70/Hsc70	[74]
Replication Protein A1 (RFA1)	Nucleus	Assists HSF1 to access nucleosomal DNA for transcription regulation of HSPs.	[24]
Nucleoporin 214 kDa (NUP214)	Nuclear membrane	Part of the nuclear pore complex. critical role in nucleocytoplasmic transport	[75]
Nuclear pore complex protein 133 kDa (NUP133)	Nuclear membrane	Part of the nuclear pore complex. critical role in nucleocytoplasmic transport	[75]
Nuclear pore complex protein 358 kDa (NUP358, RANBP2)	Nuclear membrane	SUMO1 E3 ligase. Controls the shuttling of proteins between the nuclear and cytoplasm compartments of the cell	[75]

**Fig. 2:**
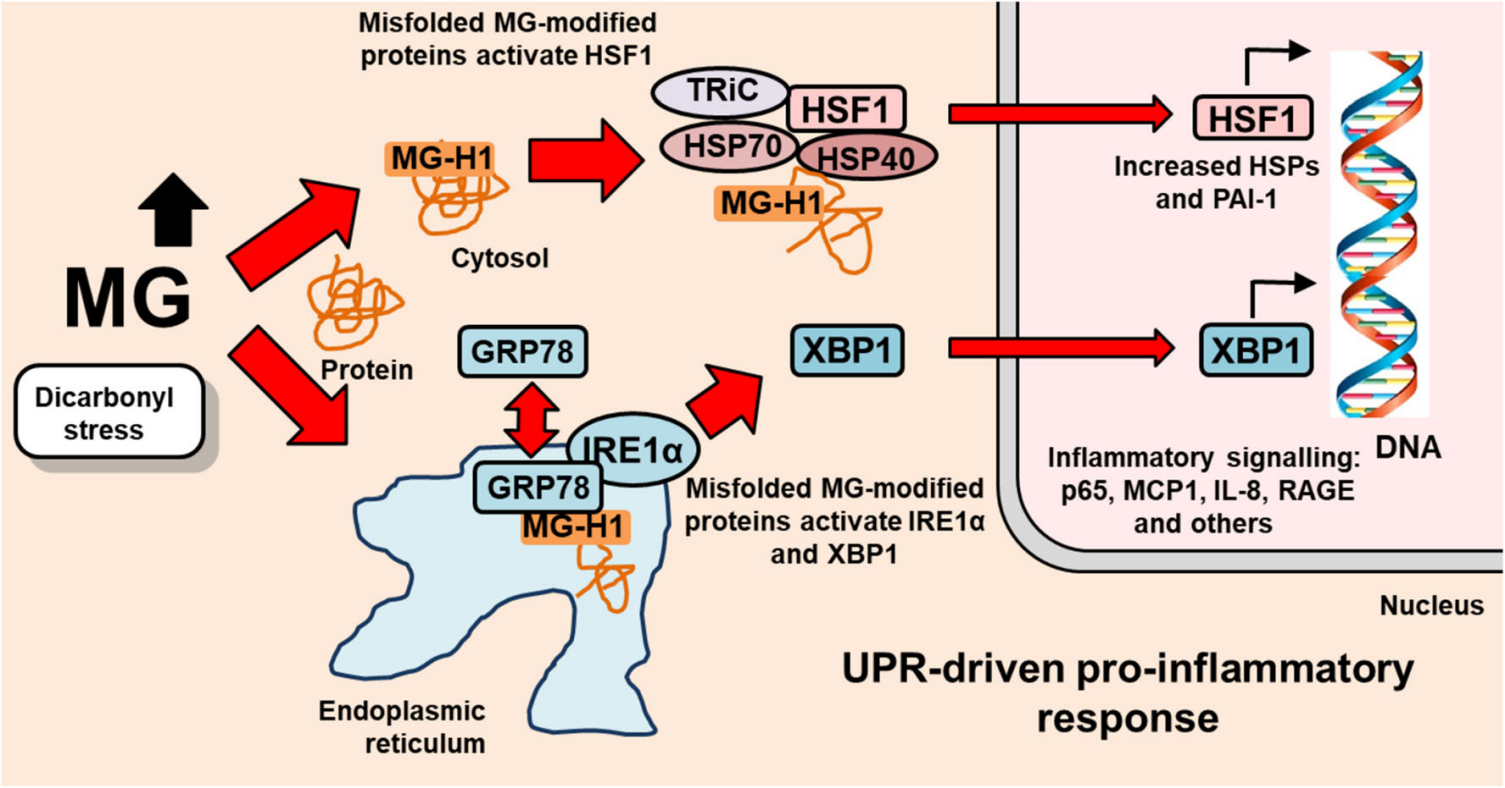
Activation of the cytosolic and endoplasmic reticulum UPR by misfolded MG-modified proteins. Schematic diagram of the mechanisms of activation of the unfolded protein responsive and pro-inflammatory response by dicarbonyl stress and increased glucose metabolism in endothelial cells in hyperglycemia. Adapted from [7]

We envisage that increased expression of HSPs reflects increased activation of HSF-1 in response to MG-driven proteotoxic stress. Inactive HSF-1 monomers complex with HSP40, HSP70, HSP90 and the chaperonin TCP-1 ring complex (TRiC) – Fig. 2. TriC chaperonins were also a target of MG modification [7]. Displacement of HSF-1 from this complex leads to its oligomerization and nuclear translocation for transactivational response, where HSF-1 has several post-translational modifications (ser and thr phosphorylation, acetylation, ubiquitin and small ubiquitin-like modifier (SUMO)) and binds to RFA1. Later, it re-associates in monomeric inactive form in the chaperonin/HSP complex [26]. MG-modified proteins are thereby likely funneled through the HSP pathway, through the sensing and binding of an unshielded, surface hydrophobic MG-H1 residue. Where MG-modified protein cannot be refolded to shield MG-H1 from the surface – we assume this is likely in most cases, they are targeted for degradation.

In the study of HAECs, in high glucose concentration and dicarbonyl stress, the abundance of 3 ubiquitin protein ligases were increased: E2/E3 hybrid ubiquitin-protein ligase UBE2O (+ 47 %); E3 ligase, HECTD1 (+ 34 %); and ligase E3C, UBE3C (+ 21 %) [7]. UBE2O acts as an autonomous quality control factor in protein homeostasis by recognition and elimination of unassembled protein subunits of protein complexes with proximate basic and hydrophobic patches on unassembled proteins [27]. HECTD1 interacts with HSP90 to regulate its cell location and degradation [28]. UBE3C improves degradation of protein fragments that arise by incomplete proteolysis of substrates by the proteasome [29]. In hyperglycemia-induced dicarbonyl stress in periodontal ligament fibroblasts (PDLFs), there was abundance of ubiquitin ligases: HUWE1 (+ 460 %), CHIP (+ 104 %) and RNF31 (+ 97 %) [8]. HUWE1 is an E3 ubiquitin ligase targeting substrates with unshielded, hydrophobic segments [30] – befitting of MG-modified proteins. Interestingly, conditional knockout of HUWE1 in pancreatic beta-cells of mice accelerated the age-dependent decline of insulin secretion and glucose homeostasis [31], which may mimic the effect of chronic dicarbonyl stress in clinical insulin resistance [32]. CHIP, as mentioned above, is a functional partner of HSP70. RNF31, also called heme-oxidized iron regulatory protein ubiquitin ligase-1-interacting protein (HOIP), is part of the linear ubiquitin chain assembly complex (LUBAC). Activation of LUBAC has a key role in proteotoxic stress, producing ubiquitin chains on misfolded proteins which wrap round it, preventing undesirable interactions which may otherwise contribute to pathogenesis [33]. The increased ubiquitination response found in dicarbonyl stress is, therefore, a response to unexpected surface hydrophobic patches on proteins and peptides, increasing their degradation and guarding against unwanted interactions such as aggregation. It is a response to change in surface physicochemical properties and related misfolding of proteins rather than a response to a specific chemical structure of the MG moiety of MG-glycated proteins (Table 2).

**Table 2:** Ubiquitin ligases increased in cellular models of dicarbonyl stress *in vitro*

Ubiquitin ligase	Function	Reference
UBE2O	Autonomous quality control factor in protein homeostasis by recognition and elimination of unassembled protein subunits of protein complexes with proximate basic and hydrophobic patches on unassembled proteins.	[27]
HECTD1	Regulates cellular location and degradation of HSP90.	[28]
UBE3C	Improves degradation of protein fragments that arise by incomplete proteolysis of substrates by the proteasome.	[29]
HUWE1	Targets substrates with unshielded, hydrophobic segments.	[30]
CHIP	Functional partner of HSP70.	[69]
RNF31 (HOIP)	Part of the LUBAC, producing ubiquitin chains on misfolded proteins which wrap round the substrate, preventing undesirable interactions contributing to pathogenesis, e.g. aggregation.	[33]

**Fig. 3:**
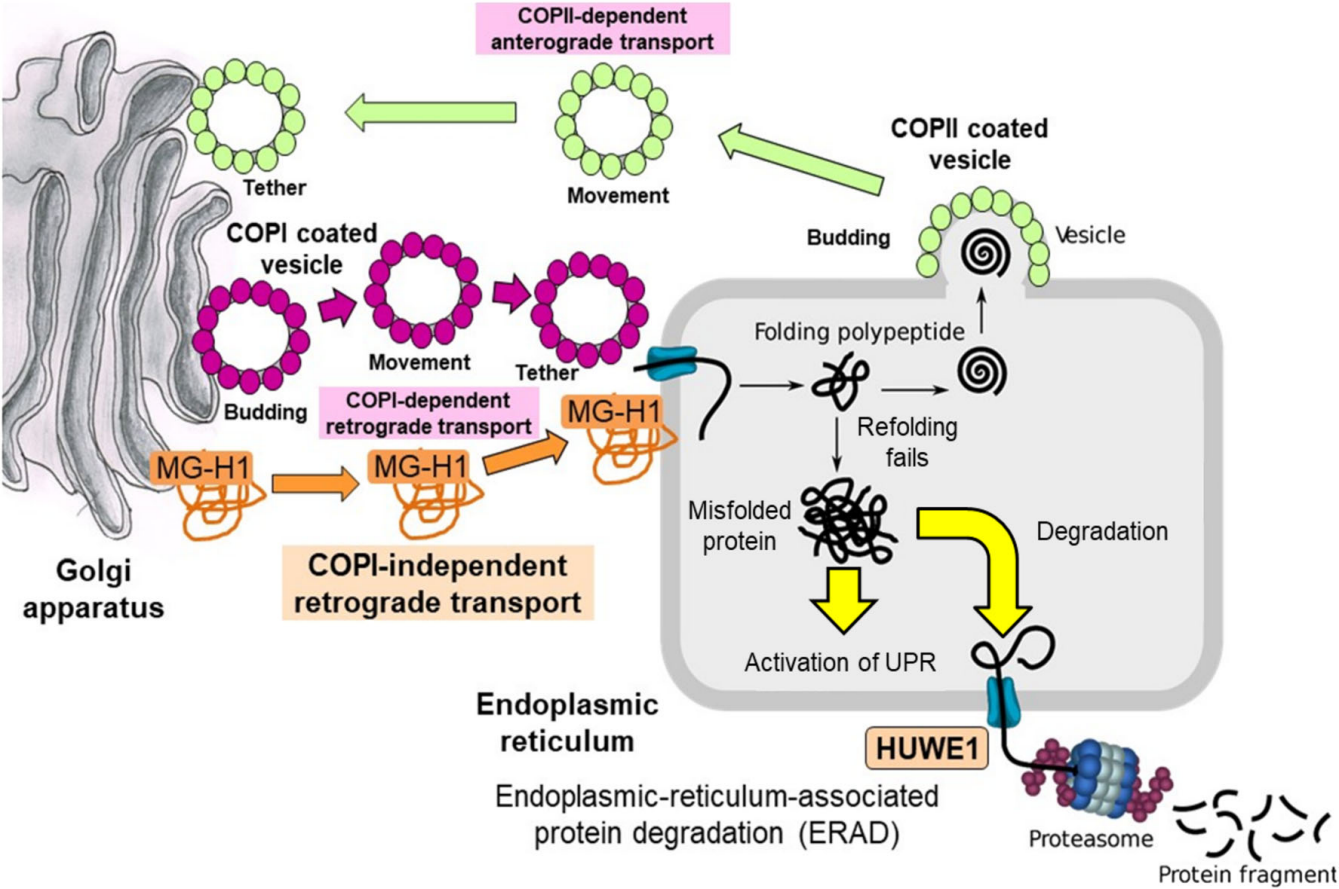
Bidirectional transport between the ER and the Golgi mediated by COPII and retrograde transport mediated by COPI-dependent -independent carriers. Schematic diagram shows budding, movement, tethering, and uncoating and fusion of COPII and COPI carriers. COPI-independent transport of MG-modified proteins likely involves vesicular transport along connecting tubules [36, 37]. After return to the ER, MG-modified proteins meet the substrate preference for ubiquitination by HUWE1 and degradation

A further remarkable finding in the study of dicarbonyl stress in PDLFs was the finding of increased Golgi-to-ER retrograde traffic of proteins. This is part of the cell response to increased misfolded proteins: misfolded proteins are returned to the ER for refolding [34, 35]. Activation of the UPR is considered to have a major effect on shuttling of proteins between the Golgi apparatus and the ER where the flux of protein cargo may influence the vesicular COPI-dependent and tubular COPI-independent retrograde protein transport [36, 37]. Repeated shuttling of proteins between the ER and Golgi apparatus is considered to be part of the quality control mechanisms to support protein homeostasis. Misfolded proteins are returned from the Golgi apparatus to ER for correction of protein misfolding; and, for covalently-modified and proteins such as MG-modified proteins where misfolding fails, proteins are diverted for proteolysis, ER-associated protein degradation (ERAD) – Fig. 3.

## Inflammatory signalling in dicarbonyl stress via the unfolded protein response

Increased low grade inflammation is a consequence of activation of the UPR. All three main pathways of the UPR, ATF6, PERK and XBP-1, have been linked to inflammatory signalling [38, 39]. A pathway where dicarbonyl stress may be particularly implicated is XBP-1 related activation of histone-lysine N-methyltransferase SETD7. XBP-1 activation induces expression of SETD7 which increases methylation of lysine 4 of histone 3 on NF-kB p65 promoter, p65 expression and binding activity. This epigenetic signal was associated with upregulation of NF-kB inflammatory system with subsequent transcription of inflammatory gene expression: increased intercellular cell adhesion molecule-1 (ICAM-1), monocyte chemoattractant protein-1 (MCP-1), receptor for advanced glycation endproducts (RAGE), interleukin-8 (IL-8) and GRP78 [40–42]. A previous remarkable finding was that this proinflammatory signaling mediated by SETD7 and activated by transient and persistent model hyperglycemia was inhibited by overexpression of Glo1 [43]. This suggests that the XBP1/SETD7 pathway of epigenetic signaling for increased low-grade inflammation may be a pathogenic consequence of activation of the UPR by dicarbonyl stress, and that the key inflammatory signaling pathway in response to dicarbonyl stress is via XBP-1.

Key to activation of XBP-1 is chaperone GRP-78 activation of the serine/threonine kinase inositol-requiring enzyme-1alpha (IRE1α). This leads to the stimulation of endoribonuclease activity of IRE1α and the splicing of XBP1 mRNA to form functionally active XBP-1. A further consequence of activation of IRE1α is decrease of micro-RNA-17, miR-17, and stabilization of thioredoxin interacting protein (TXNIP) mRNA and increased expression of TXNIP [44]. TXNIP decreases glucose uptake by skeletal muscle and pancreatic beta-cell mass and insulin secretion and increases hepatic gluconeogenesis. It is thereby a major mediator of insulin resistance [45–47]. This and related UPR stimulated low grade inflammation likely contributes to pathogenesis in insulin resistance, obesity, type 2 diabetes mellitus (T2DM), non-alcoholic fatty liver disease (NAFLD), chronic kidney disease, cardiovascular disease and age-linked decline of respiratory function [48–58] – Fig. 2.

## Glyoxalase 1 inducer therapeutics

The above coverage provides evidence and argument for dicarbonyl stress providing physiological substrate, MG-modified proteins, for the UPR. With this potential advance in understanding of pathogenic mechanism, it is important to identify and develop pharmacological mechanisms to exploit the advance for effective intervention with novel drug therapy. An effective and efficient strategy to counter dicarbonyl stress is development of Glo1 inducers. Glo1 inducers increase Glo1 expression and activity, correct increased MG concentration and formation of MG glycated proteins, and thereby cut off activation of the UPR at source. Glo1 inducers have important advances over other potential pharmacological strategies to decrease MG – as reviewed [32]. We identified an optimum Glo1 inducer available from dietary bioactive compounds which activate transcription factor Nrf2. GLO1 is an antioxidant response element (ARE) linked gene with basal and inducible expression regulated by Nrf2 [59]. We screened individual and synergistic binary combinations of dietary bioactives compounds for induction of Glo1 expression using a GLO1-ARE luciferase reporter assay. The optimum Glo1 inducer was a combination of *trans*-resveratrol and hesperetin, tRES-HESP. Induction of Glo1 expression was validated at mRNA and protein levels in human cell cultures – primary cultures of HAECs and BJ fibroblasts, and the hepatocyte-like HepG2 cell line [5].

For clinical evaluation, we performed a double-blind, randomised, placebo-controlled crossover study in overweight and obese subjects with 8 weeks treatment with tRES-HESP; one capsule daily before breakfast containing 90 mg tRES and 120 mg HESP. For target pharmacology, we found increased expression and activity of Glo1, decreased plasma MG concentration and decreased total body flux of formation of MG-H1. In evaluation of effects on dysglycemia and insulin resistance, we found decreased fasting plasma glucose (FPG) and postprandial plasma glucose and correction of insulin resistance by tRES-HESP; placebo had no effect. We also found improved arterial dilatation and decreased vascular inflammation marker, soluble ICAM1 [5]. Comparison of the effect of tRES-HESP in overweight and obese subjects to that of metformin and Orlistat – potential alternative treatments targeting other pathogenic mechanisms - on similar subjects groups in intervention trials suggested the effect of tRES-HESP on glycaemic control exceeds that of metformin and matches that of Orlistat [60, 61]. The improvement in insulin resistance is comparable to that achieved with extreme weight loss with gastric band surgery in morbid obesity [62]. tRES-HESP may also improve the function of pancreatic beta-cells [5]. Decrease in FPG in the normal range is associated with reduced risk of developing T2DM [63]. The Glo1 inducer formulation could be a suitable treatment for improved metabolic and vascular health in overweight and obese populations [5]. Insulin resistance is also linked to NAFLD, chronic kidney disease, cardiovascular disease, decline of respiratory function and aging [48–58] – likely through increased low grade inflammation linked to hyperinsulinemia [64]. With a safe and effective treatment of insulin resistance, prevention and early-stage reversal of T2DM and other insulin resistance linked pathology and mortality may be improved. There is currently no pharmacotherapy specifically targeting mechanisms of insulin resistance - although several pharmacological approaches are in development [65, 66]. Glo1 inducers, therefore, offer an improved, safe and effective route to novel insulin sensitizing agents [5].

## Concluding remarks

The UPR plays a vital role in proteostasis through surveillance of quality of the proteome, sensing damaged and misfolded proteins and activating processes of protein refolding for repair or proteolysis for removal [67]. With recent studies on physiological dicarbonyl stress and identification of the proteomic response to it, protein glycation by MG has emerged as a potential key physiological activator of the UPR. Further investigation and application of Glo1 inducer therapeutics are required. If confirmed, key advances in understanding of physiological substrates and activators of the UPR in aging and disease may emerge where increase of Glo1 expression may be a key pharmacological target.

## Abbreviations

AGE: Advanced glycation endproduct
ApoB100: Apolipoprotein B100
ARE: Antioxidant response element
ATF6: Activating transcription factor 6
COPI: Coatomer protein I
COPII: Coatomer protein II
ER: Endoplasmic reticulum
ERAD: Endoplasmic reticulum-associated protein degradation
FACT: Facilitates chromatin transcription
FL: N_ε_-(1-Deoxy-D-fructos-1-yl)lysine
FPG: Fasting plasma glucose
Glo1: Glyoxalase 1
Glo2: Glyoxalase 2
GRP78: Glucose regulated protein 78 kDa
GSH: Reduced glutathione
HAECs: Human aortal endothelial cells
HDL: High density lipoprotein
HOIP: Heme-oxidized iron regulatory protein ubiquitin ligase-1-interacting protein
HSF-1: Heat shock factor-1
HSP: heat shock protein
ICAM-1: Intercellular cell adhesion molecule-1
IL-8: Interleukin-8
IRE1α: Inositol-requiring enzyme-1alpha
LDL: Low density lipoprotein
LUBAC: Linear ubiquitin chain assembly complex
MCP-1: Monocyte chemoattractant-1
MG: Methylglyoxal
MG-H1: N_δ_-(5-Hydro-5-methyl-4-imidazolon-2-yl)ornithine
NAFLD: Non-alcoholic fatty liver disease
Nrf2: Nuclear factor erythroid 2–related factor 2
NUP133: Nuclear pore complex protein 133 kDa
NUP214: Nucleoporin 214 kDa
NUP358: nuclear pore complex protein 358 kDa
PDLF: Periodontal ligament fibroblast
PERK: Protein kinase RNA-like endoplasmic reticulum kinase
RAGE: Receptor for advanced glycation endproducts
RANBP2: Ras-related nuclear binding protein 2
RFA1: Replication protein A1
SETD7: Histone-lysine N-methyltransferase
SUMO: Small ubiquitin-like modifier
T2DM: Type 2 diabetes mellitus
tRES-HESP: *trans*-Resveratrol and hesperetin combination
TRiC: Chaperonin T-complex protein-1 ring complex
TXNIP: Thioredoxin interacting protein
UPR: Unfolded protein response
VLDL: Very low density lipoprotein
XBP-1: X-box binding protein 1
